# Global health in the 21st century

**DOI:** 10.3402/gha.v7.23694

**Published:** 2014-02-13

**Authors:** Ulrich Laaser, Helmut Brand

**Affiliations:** 1 World Federation of Public Health Associations, c/o IGH/CMU, University of Geneva, Geneva, Switzerland; 2 Faculty of Health Sciences, University of Bielefeld, Bielefeld, Germany; 3 Association of Schools of Public Health in the European Region (ASPHER), Brussels, Belgium; 4 Department of International Health, CAPHRI School for Public Health and Primary Care, Faculty of Health, Medicine and Life Sciences, Maastricht University, Maastricht, The Netherlands

**Keywords:** global health, health equity, key challenges, emerging global structures, civil society, global public health terminology

## Abstract

**Introduction:**

Since the end of the 1990s, globalization has become a common term, facilitated by the social media of today and the growing public awareness of life-threatening problems common to all people, such as global warming, global security and global divides.

**Review:**

For the main parameters of health like the burden of disease, life expectancy and healthy life expectancy, extreme discrepancies are observed across the world. Infant mortality, malnutrition and high fertility go hand in hand. Civil society, as an indispensable activator of public health development, mainly represented by non-governmental organisations (NGOs), is characterised by a high degree of fragmentation and lack of public accountability. The World Federation of Public Health Associations is used as an example of an NGO with a global mission and fostering regional cooperation as an indispensable intermediate level.

The lack of a globally valid terminology of basic public health functions is prohibitive for coordinated global and regional efforts. Attempts to harmonise essential public health functions, services and operations are under way to facilitate communication and mutual understanding.

**Recommendations:**

1) Given the limited effects of the Millennium Development Goal agenda, the Post-2015 Development Goals should focus on integrated regional development. 2) A code of conduct for NGOs should be urgently developed for the health sector, and NGOs should be registered and accredited. 3) The harmonisation of the basic terminology for global public health essentials should be enhanced.

The 21st century began with the first truly global effort to enhance the health of the people in all parts of the world until the year 2015: The Declaration of the Millennium Development Goals (MDGs) in 2000 (1). Improved health, especially for mothers and children, together with the elimination of the most ravaging infectious diseases – malaria, HIV/AIDS and tuberculosis ȁ3 was embedded into a set of social goals, especially reduction of poverty, as well as structural ones like the development of effective partnerships, thus acknowledging the interrelationship of health and its major determinants. After 2015, efforts by the United Nations to achieve a world of prosperity, equity, freedom, dignity and peace will continue unabated (2) and very likely focus even more on social and structural goals. The only comparable endeavour before 2000 was the Declaration of Alma-Ata in 1978 (3) calling for the implementation of primary health care (PHC) throughout the world. However, more than three decades later, it cannot be said that PHC has achieved the lofty vision and objectives delineated in Alma-Ata (4) although the concept has been widely accepted. A major breakthrough was also the report of the Commission on Social Determinants of Health (5) followed by an inestimable number of affirmative papers and statements. Since its publication in 2008, the achievement of equity in health is unquestioned as an objective.

Since the end of the 1990s, globalization has become a common term, facilitated by the social media of today and the growing public awareness of life-threatening problems common to all people, such as global warming, global security and global divides. Health equity challenges are reflected in unequal burdens of disease and grossly unequal means to improve the population's health (6). The contributions to this special issue of *Global Health Action* attempt to cover the most important threats and opportunities of global health in the new century. Our paper concentrates on three key issues if to add a global dimension to the ‘new public health’ concept: 1) the gross inequity of the global burden of disease (BoD); 2) the growing global involvement of innumerable non-governmental organisations (NGO) for health; and 3) the present efforts to globally harmonise basic terminology in order to facilitate communication between the multiplicities of public health actors. These three issues are closely interconnected and central determinants of the future of global public health in the 21st century. The North–South gradient of BoD is unacceptable from any moral point of view but without the engagement of the civil society, predominantly organised as NGOs, change will be hard to achieve. This poses a communication problem, namely between the language of the civil society and that of formally trained public health professionals. We shall review the three fields and finally integrate the information in terms of conclusions.

## The global state of health

**Fig. 1:**
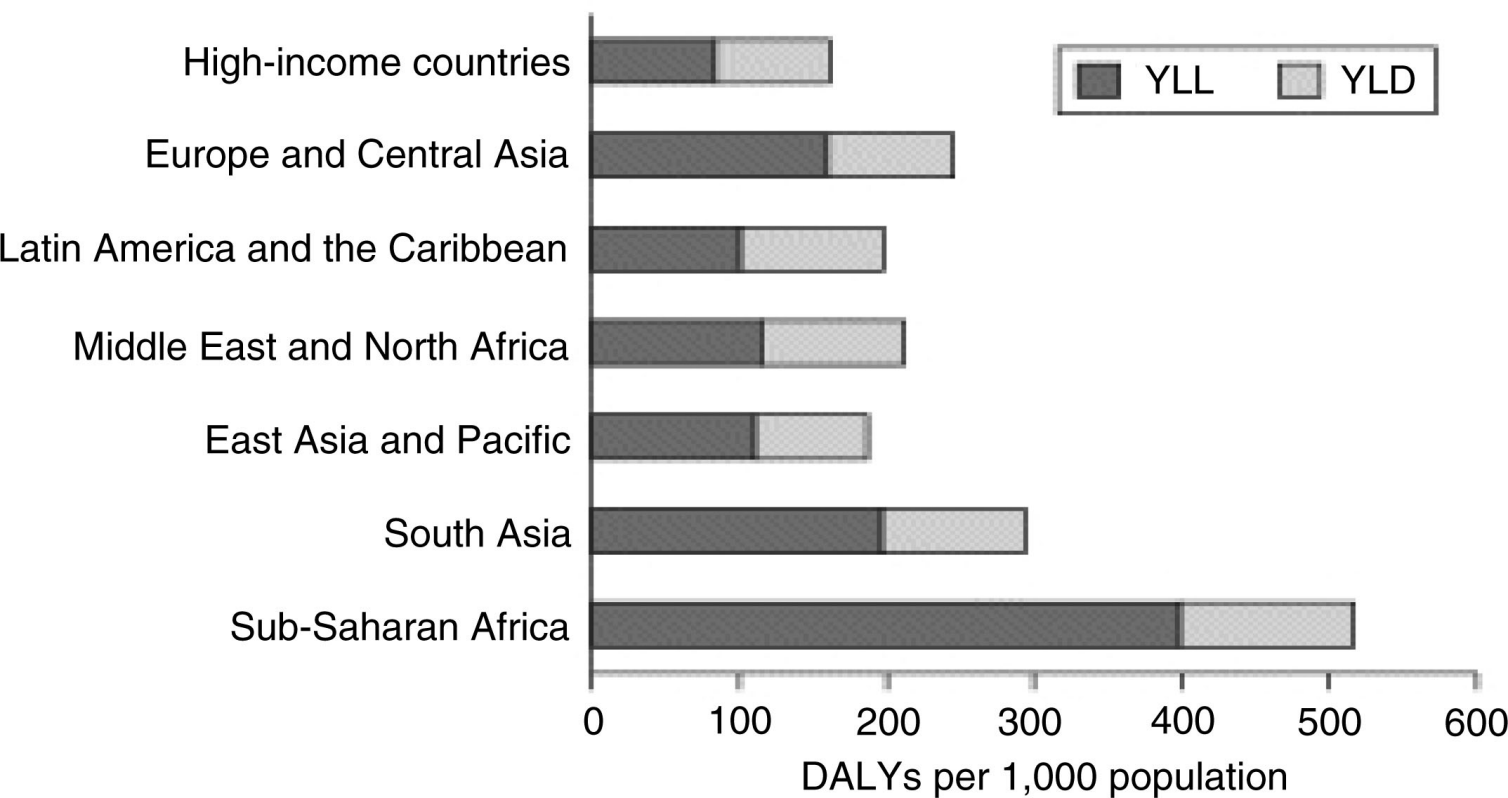
Years of life lost (YLL), years lost to disability (YLD) and disability-adjusted life years (DALYs) by region (2001) (from reference (9)).

Estimates of the BoD, a technique developed mainly by Murray and Lopez (e.g. 7, 8), constitute the most important, although not exclusive, measure to compare populations’ health across the globe in terms of a global BoD. The BoD is usually expressed in the form of years of life lost (YLL) in comparison to a set optimum (Japanese women) and years lost to disability (YLD), summed up together as disability-adjusted life years (DALYs) lost. Fig. 1 shows the global distribution of these parameters: in high-income countries we find less than 200 DALYs/1,000 population, in sub-Saharan Africa 500, and an even greater contrast of about 4:1 for YLL considered separately (9).

In terms of life expectancy (LE) at birth, this means that the average for Africa is 54 vs. 75 years in Europe (in 2009), although LE in Africa increased by 3 years from 1990 to 2009, i.e. from 51 to 54 (see Table 1) (10). Throughout these two decades, Japan has remained at the top for both sexes, whereas the last positions are taken by African countries, joined in 2010 by Haiti (11). Nevertheless, the gap between countries with the longest and shortest total LE is slowly closing.

**Table 1:** Life expectancy at birth, both sexes (males/females)

WHO region	1990	2000	2009
Africa	51 (49/53)	50 (48/52)	54 (52/56)
Americas	71 (68/75)	74 (71/77)	76 (73/79)
South-East Asia	59 (58/59)	62 (61/64)	65 (64/67)
Europe	71 (68/75)	72 (68/76)	75 (71/79)
Eastern Mediterranean	61 (59/63)	64 (62/65)	66 (64/67)
Western Pacific	69 (68/71)	72 (70/74)	75 (72/77)

Recently the research group around Murray and Lopez published an analysis of healthy life expectancy (HALE) in the *Lancet* (12). The point estimates of HALE at birth increased between 1990 and 2010 globally by 4.2 years for males and 4.5 for females; even at the age of 60 years, the gains are still estimated as 0.7 and 0.8, respectively.

The gap between countries occurs for very different reasons, if one looks at YLL according to communicable diseases (CD), injuries and non-communicable diseases (NCD) (13). The relation NCD/CD is, e.g. 0.19 for Africa vs. 6.5 for Europe, while globally almost balanced at 0.8. These relations correspond in an almost identical way to the calculated ratios of World Bank income groups, ranging from the lowest quartile to the highest (14). Salomon et al. (12) predict a changing balance for Africa; projecting that by 2060 the ratio NCD/CD will be also around five, similar to today's for Europe.

**Fig. 2:**
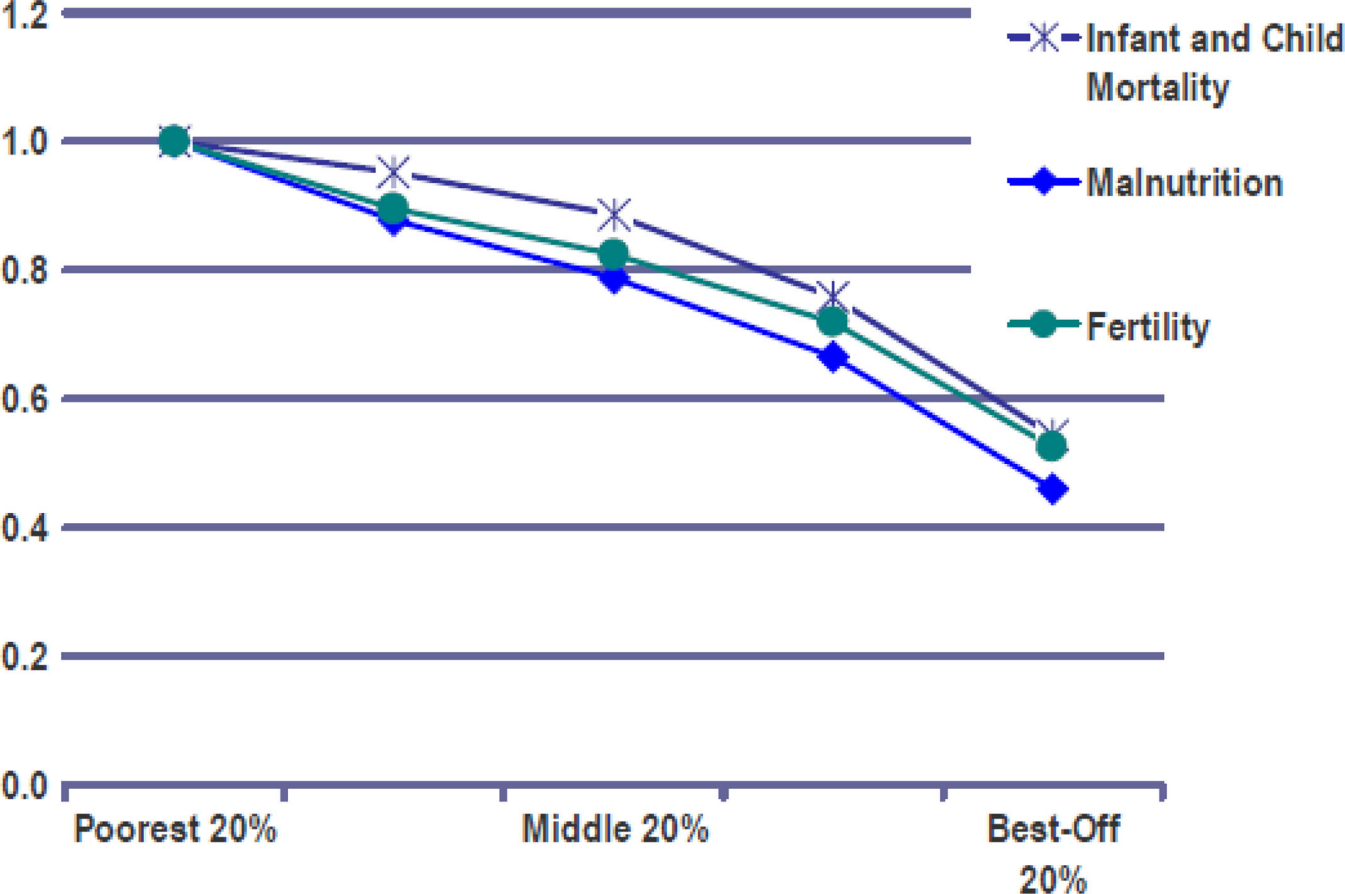
Economic inequalities with respect to selected indicators of health, nutrition and population status (17) (the findings are expressed in relative terms, with the level prevailing in the poorest quintile set at 1.0).

Furthermore, the disparities regarding HALE are related to demographic development. Although the growth rate of the world population has reduced from its peak of 2.2% per year in the early 1960s to around 1.5% now (15), most of the present population growth is taking place in the poorest developing countries. Fertility rates are decreasing in all regions, even in Africa from almost seven children per woman in the early 1950s to a still high level of around five at the end of the first decade of this century (16). The differing growth rates are linked to vast disparities in wealth, health and opportunities (17). Figure 2 shows how closely the social gradients for infant and child mortality as well as malnutrition are linked with fertility. Table 2 exemplifies this in more detail for selected health parameters, comparing the lowest and the highest social quintile around the turn of the century. For example, the quotient of highest/lowest quintile reaches 1.4 (measles vaccination), 1.6 (medical treatment of respiratory infection) and 2.4 (medically attended delivery).

**Table 2:** Selected vaccinations, diseases and perinatal parameters for 56 developing countries (adapted from (17)

	Lowest quintile	Highest quintile	Average
BCG coverage	72.0	92.4	81.3
Measles coverage	57.5	81.8	68.0
DPT coverage	53.0	77.8	63.7
Diarrhoea	19.0	13.9	17.2
Oral rehydration	56.2	70.7	69.9
Respiratory infection	16.9	13.6	16.0
Medical treatment	36.4	59.7	46.1
Contraceptive prevalence, women	20.2	38.7	28.7
Antenatal care visits to a medically trained person	62.1	92.6	76.1
Delivery attended by a medically trained person	35.8	85.0	55.3

In 1978, the Alma-Ata Declaration already concluded that ‘The existing gross inequality in the health status of the people particularly between developed and developing countries as well as within countries is politically, socially and economically unacceptable’ and ‘Governments have a responsibility for the health of their people which can be fulfilled only by the provision of adequate health and social measures’ (3). One estimate of the combined cost of health inequities and premature death in the United States alone amounts to $1.24 trillion between 2003 and 2006 (18). Laaser and Epstein (6) defined three main barriers, namely barriers to equitable access, financial barriers and cultural barriers.

## The growing role of NGOs

NGOs or civil society organisations (CSO) play a strong role in the health sector. In 2006, for example, almost 25% of the total development assistance for health (DAH) was channelled through NGOs, almost a doubling of the share of 13.1% in 1990 (19), and more than through the global health initiatives (GHI), where the Global Fund, GAVI and the Bill & Melinda Gates Foundation together amount to 16.4%. This has also been called the ‘new dichotomy’ in health systems development (20). Aid through bilateral channels still accounts for around one third of all DAH (21). Poor countries account for 56% of the global disease burden but less than 2% of global health spending (22). In 2009, Piva and Dodd (23) analysed the flow of resources through official development assistance (ODA). They revealed that almost one third of total ODA for health between 2002 and 2006 went to HIV/AIDS, thereby contributing a large share of the increases in health ODA. Furthermore, a substantial part of health ODA was spent on technical cooperation, comprising grants to nationals for education or training, payments for consultants and administration, and for equipment. Technical cooperation made up 41.7% of all health ODA and only 58.6% was channelled through multi-country initiatives. As shown by Piva and Dodd (23), aid is highly fragmented at country level with unnecessary transaction costs, disruption of national policies and lack of coordination. Between 2002 and 2006, the OECD's aggregate aid statistics and creditor reporting system (CRS) recorded 20.485 health projects, among them many very small projects, i.e. 13.819 accounting for only 3.6% of total health ODA. Some remedies to overcome this fragmentation have been discussed, including direct budget support and a sector-wide approach (SWAp) (6, 24), but up to now seemingly these concepts did not gain larger relevance: only 6.4% of total ODA went to direct budget support and 7.7% of health ODA to sector programmes (23).

If one looks at the progress on the MDGs, it appears that the goal of 21% living below the poverty line defined as 1.25 USD/day was within reach in 2005. However, this was calculated from a baseline set at 1990, i.e. a decade before the MDGs were declared. If one compares the progress between 1990 and 1999 of 11 percentage points to the progress between 1999 and 2005 of 6 percentage points then it becomes apparent that the pace of development has been quite similar before and after the MDG commitment in the year 2000. In addition, the largest chunk of progress is due to the over-achievement of China, not only halving but quartering its poorest population (6). Similarly, one could take malnutrition as another indicator with 19.8% in the developing countries in 1990 coming down to 16.8 in 1995 and remaining stagnant at 15.5% in 2006. However, the sheer numbers of malnourished remain stable at 848 million in 1990 vs. 850 in 2008. In sub-Saharan Africa, the numbers even increased in the last period (2003–2008) from 211 to 231 million (25). Reduction of maternal mortality shows the same overall pattern, coming down from 440/100,000 live births in 1990 to 350 in 2000 and 240 in 2010, i.e. with almost identical decrease in each of the two decades. It seems that their global answers succeed only half way as Ban Ki-Moon, Secretary General, United Nations stated in 2012: ‘Projections indicate that in 2015 more than 600 million people worldwide will still be using unimproved water sources, almost 1 billion will be living on an income of less than $1.25 per day, mothers will continue to die needlessly in childbirth, and children will suffer and die from preventable diseases’ (25) and he continued: ‘Achieving the MDGs by 2015 is challenging but possible. Much depends on the fulfilment of MDG-8 – the global partnership for development’. It seems that voluntary partnership is not enough vis-a-vis a reality which is characterised by ‘… unevenness of progress within countries and regions, and the severe inequalities that exist among populations, especially between rural and urban areas’ (ibidem).

The paradigm of global health aid is shifting away from governmental agencies towards the civil sector. NGOs and foundations make up by now a large piece of health assistance, but there are serious imbalances between DAH and the BoD as shown by Ravishankar et al. in 2009 (19). DAH for the last decade obviously has been given not according to the highest disease burdens, which are found in South Asia and sub-Saharan Africa (9), but other criteria as e.g. political and economic interests. Whereas governments are at least to some degree responsible to their population, NGOs and foundations are responsible to their constituency but not always do they feel such obligation towards their clientele and the general public. They usually act on their own and cooperate only on a case-by-case basis. Certainly a strong role of NGOs and GHIs constitutes an essential contribution, but the resulting fragmentation and lack of coordination together with a deficit in accountability (24) as well as the described financial imbalances questioning why that is and what could be done about it. Obviously, some structural element is missing between the global agenda and a more successful implementation of well-designed strategies and invested funds. Are the still-weak global structures able to direct implementation? Frenk (26) already asked these questions in 2010 and argued that national health systems must be strengthened in order to progress globally. But will smaller countries ever be able to assemble the diversity of skills required? Will even larger ones provide the long-term stability to guarantee permanent progress?

To overcome some of the above challenges, it may be valuable to strengthen regional cooperation as a priority, not only on a voluntary basis but organised as long-term binding agreements. Well-established examples are the European Union (EU) (27), the Association of Southeast Asian Nations (ASEAN) and the African Union (AU). Whereas the EU has defined a political mandate for the European Commission in the Maastricht treaty of 1993, ASEAN has defined ‘consultation and consensus’ as one of its key principles under the umbrella of the ‘ASEAN way’. The AU together with WHO-AFRO supported strongly the formation of the African Federation of Public Health Associations (AFPHA) in 2011 (28), being a model of the new regionalization strategy of the World Federation of Public Health Associations (WFPHA) (29).

The role of the WFPHA is described here as an example of how NGOs can contribute in a meaningful way to global health development. The WFPHA is the only non-governmental global body representing public health professionals and their national organisations. It is officially registered with the World Health Organization, and plays a leadership role in the quest for a healthy global society comprising the right to health, diversity and inclusion, partnership and ethical conduct (30). The WFPHA advocates for a strong civil society voice, the active participation of national public health associations, allied groups in national and global discussions and decision-shaping around public health policy and practice (31).

The creation of the aforementioned AFPHA started in a climate of support and cooperation between all African Public Health Associations unseen before, supported by the African office in Addis Ababa, Ethiopia, and with a first African Conference organised in Cape Town in September 2013 (32). Parallel to the changing social and political environment in the Eastern Mediterranean region, also a 1st Arab World Conference on Public Health was organised in Dubai, in early April 2013 (33). The 4th common conference on public health in the Western Pacific arena took place in Na Trangh, Vietnam, in November 2013 (34). This only became possible through the foundation of the Asian Pacific Regional Liaison Office in Beijing in 2009. Public health constitutes a neutral terrain and enables the opening of doors which otherwise would be kept closed. A good example is the intensive post-war collaboration developed between the successor states of the former Yugoslavia and their neighbours (35) in the framework of the European Stability Pact. All regional cooperation developed in the realm of the WFPHA as described is based on formalised arrangements within WFPHA. It is believed that such formalisation and mutual obligation at the regional level is the missing link towards the more effective and more efficient implementation of health aid channelled through NGOs as discussed above. Therefore, mechanisms of integration like the SWAp and the discussion around reform of WHO, broadening the decision-making process by a so-called ‘Committee C’ to involve NGOs (21) are seen as mechanisms to strengthen the role of NGOs whilst simultaneously holding them more accountable for their activities.

## A common language for global health?

Health services as well as health financing systems are governed nationally and therefore vary considerably between countries. Nevertheless, since the time of Bismarck's inauguration of a German health insurance system in 1883 and the start of the National Health Service (NHS) in the United Kingdom in 1948 (based on the Beveridge Report of 1940), in the last two decades we observe increasingly similar trends worldwide; for example, in the former communist countries in Eastern Europe one can find elements of the so-called Bismarck and Beveridge systems in various combinations [‘Bisridge’ systems, e.g. (36)]. Related is a growing need for common terminologies especially in the public health arena. In this context a global dialogue on standardised lists of professional competences for public health started towards the end of the last century. However, so far there is only partial agreement on the underlying public health functions that a well performing health system should deliver and for which the workforce should be trained. The lack of a common vocabulary in public health is prohibitive for coordinated global efforts.

Core public health functions may be considered as a set of fundamental activities that address determinants of health, and maintain and protect population health through organised efforts of society. There are several lists of public health functions and services or operations that exhibit differences which can hardly be justified by arguments. In a broader sense, this situation signifies that there is yet insufficient agreement on what is public health, what it does and whether it acts effectively.

Table 3 shows three prevailing sets of regional core functions: the Essential Public Health Functions (EPHF) as published by Pan-American Health Organization (PAHO) (37), Centers for Disease Control and Prevention (CDCs) Essential Public Health Services (EPHS) (38, 39) and the Essential Public Health Operations (EPHO) of WHO-EURO, adopted only recently (40). For comparison with the most widely used PAHO functions, the other examples are rearranged to fit as far as possible with the EPHF, however the numbers of their original sequence are maintained (41). Similar sets of basic characteristics for improving public health are missing, especially for the African continent. While the headline terminology differs in terms of functions, services and operations, as well as in the total number of items, also the functions listed are not always analogous. Although all three sets start with monitoring/surveillance and end similarly with research, there are some interesting differences.

**Table 3:** Comparison of original numeration in the lists of PAHO's Essential Public Health Functions, CDC's Essential Public Health Services and WHO-EURO's Essential Public Health Operations

PAHO's Essential Public Health Functions (EPHF)	CDC's Essential Public Health Services (EPHS)	WHO-EURO's Essential Public Health Operations (EPHO)
1. Monitoring, evaluation and analysis of health status	1. Monitor health status to identify community health problems	1. Surveillance of population health and well-being
2. Public health surveillance, research and control of risks and threats to public health	2. Diagnose and investigate health problems and health hazards in the community	2. Monitoring and response to health hazards and emergencies
3. Health promotion	4. Mobilise community partnerships to identify and solve health problems 5. Develop policies and plans that support individual and community health efforts	4. Health promotion, including action to address social determinants and health inequity
4. Social participation in health	3. Inform, educate and empower people about health issues	9. Advocacy, communication and social mobilisation for health
5. Development of policies and institutional capacity for planning and managing public health 6. Strengthening of institutional capacity for planning and management in public health 7. Evaluation and promotion of equitable access to necessary health services 9. Quality assurance in personal and population-based health services	9. Evaluate effectiveness, accessibility and quality of personal and population-based health services 7. Link people to needed personal health services and assure the provision of health care when otherwise unavailable	6. Assuring governance for health and well-being 8. Assuring sustainable organisational structures and financing
8. Human resource development and training in public health	8. Assure a competent public and personal health care workforce	7. Assuring a sufficient and competent public health workforce
10. Research on public health	10. Research for new insights and innovative solutions to health problems	10. Advancing public health research to inform policy and practice
11. Decreasing emergences and disasters in health including prevention, mitigation, preparedness, response and rehabilitation	–/–	–/–
–/–	6. Enforce laws and regulations that protect health and ensure safety	3. Health protection including environmental, occupational, food safety and others
–/–	–/–	5. Disease prevention, including early detection of illness

For example, governance is spelled out by at least four functions in the PAHO set (EPHF 5, 6, 7 and 9) but less differentiated in the CDC and EPHO set. Conversely, sustainable financing is mentioned only in the EPHO set (EPHO 8). Accessibility of personal health services is mentioned under EPHF 9 and EPHS 7, but is not found among the EPHOs. The PAHO function 11 on preparedness is not identifiable as a separate (i.e. important) function in either of the other two examples. Contrary to this, all others mention health protection and law enforcement, which one cannot find among the EPHF. Prevention is a separate operation in the WHO-EURO set (EPHO 5) but not mentioned elsewhere, whereas health promotion appears in all three sets. The sub-objectives under each function/service or operation cover most of the missing areas, but this further triggers the question as to whether the differences in terminology and order can be justified by regional or national differences regarding population health and health systems.

As different as the core functions are, there is also great variability in the list of public health competences developed all over the world (42). Among the most developed examples are the lists developed by the Association of Schools of Public Health in the European Region (ASPHER) on core competences for health professionals (43) and for the education of masters of public health (44).

In the context of ASPHER's European Public Health Core Competences Programme, the competence concept includes knowledge as well as skills. Competences should also be concretely observable/measurable, so that performance at any of these levels can be evaluated with relative ease. The subdivisions are based on the main structure of the field, i.e. the science and art of public health:

1. Methods in public health;
2. Population health and its social and economic determinants;
3. Population health and its material – physical, radiological, chemical and biological – environmental determinants;
4. Health policy; economics; organisational theory and management;
5. Health promotion: health education, health protection and disease prevention;
6. Ethics.

The authors argue that the structure in itself reflects the inter-disciplinarity of the sciences involved in public health and in principle, it is intended to be exhaustive and comparable especially to the list of core competences as developed by the Association of Schools of Public Health (ASPH) in the USA (33).

Whereas the core functions and main components of professional competence should be aligned in a global discussion process (45), this might be less meaningful for the subsequent areas of public health education (46) and of service performance (47), as they are mainly determined by national governmental and academic authorities, although to a lesser degree in Europe. In Europe, all governments have signed the Bologna Declaration (48) attempting to harmonise academic curricula and degrees with the intention to facilitate mutual acknowledgement and mobility of students and lecturers across national borders. However, the most relevant partners, the future employers of public health graduates, are usually not part of the dialogue. In Europe, they were questioned systematically for the first time in 2012 (49).

## Conclusions

We tried to cover some key issues determining global health in the 21st century. The gross imbalances are obvious and relate especially to Africa and South-East Asia. Although there is progress as regards the MDGs, the improvements are limited in the poorest countries and usually are averaged including, e.g. the over-achievement of China. To some degree, the results correspond to a straight line drawn from the early 90s through the year of the MDGs declaration in 2000 and up to the present. In other words, major additional effects are not visible. Another key determinant is the demographic pattern where fertility, malnutrition and child mortality are closely correlated.

After this rather critical review of the health status of the world today and the rather modest attempts so far to remedy it, we tried to develop two concepts that we see as preconditions for lasting change. First, we are convinced that regional cooperation across national borders is one key element to avoid military conflict with its huge toll of life and disability as well as scarce material resources, and to foster mutual exchange of knowledge, experience and opinion. At least for smaller resource-poor countries, this would increase their chances for stable, shared and forward development. A regional mechanism involving mutual control of donations could help to minimise waste, fraud and corruption and improve the chances of development projects to succeed. Therefore, the model of the SWAp, as briefly mentioned above, may be further developed in the framework of a wider regional cooperation and coordination. So far, a defined and globally authorised (by WHO, WB, IMF, GHIs?) regional strategy for health development is not available, although there are some hesitant steps in the right direction (50). In addition, closer more formalised regional cooperation between countries would also allow for a better surveillance system on the work of NGOs. Their engagement, as valuable as it might be, should be integrated into regional and national health development plans, and they have to be held publicly accountable for their financial flows and project outcomes.

Civil society has to play a vital role in confronting the real challenges of the 21st century. Many opportunities have been missed, after an abundance of assessments and analyses since the ending of the cold war in the late 1980s. As has been said in 2010 (6): ‘We have to depart from the old thinking of the 20th century, still concerned with diplomatic, economic and military power plays, and face the real challenges: the warming climate, the global divides (e.g. population growth, social and health inequity, migration, trade, security) and the missed opportunities (e.g. the recognition of health as a basic human right, the MDGs, the implementation of the Alma-Ata Declaration on PHC, the improvement of global aid mechanisms, the strengthening of good governance). This will not become possible without a strong involvement of the civil society. However, NGOs should not only be accountable to their clientele, but also to society in general’.

Second, a common terminology or language is a precondition for effective communication. We take the example of the differing arrangements of EPHF, services or operations. It would be understandable that certain issues have more relevance in one country than in another. But the framework and order should refer in the same way to the same main problems all over. Therefore, the WFPHA has established a Working Group on Public Health Professional's Education and Training (51) processing the global harmonisation of essential public health communication as a first step.

## Recommendations

The review information and the conclusions presented lead the authors to the following three key recommendations:

1. Given the limited effects of the MDG agenda, the Post-2015 Development Goals should focus on integrated regional development.
2. A code of conduct for NGOs should be urgently developed for the health sector and NGOs be registered and accredited.
3. The harmonisation of the basic terminology for global public health essentials should be enhanced.
